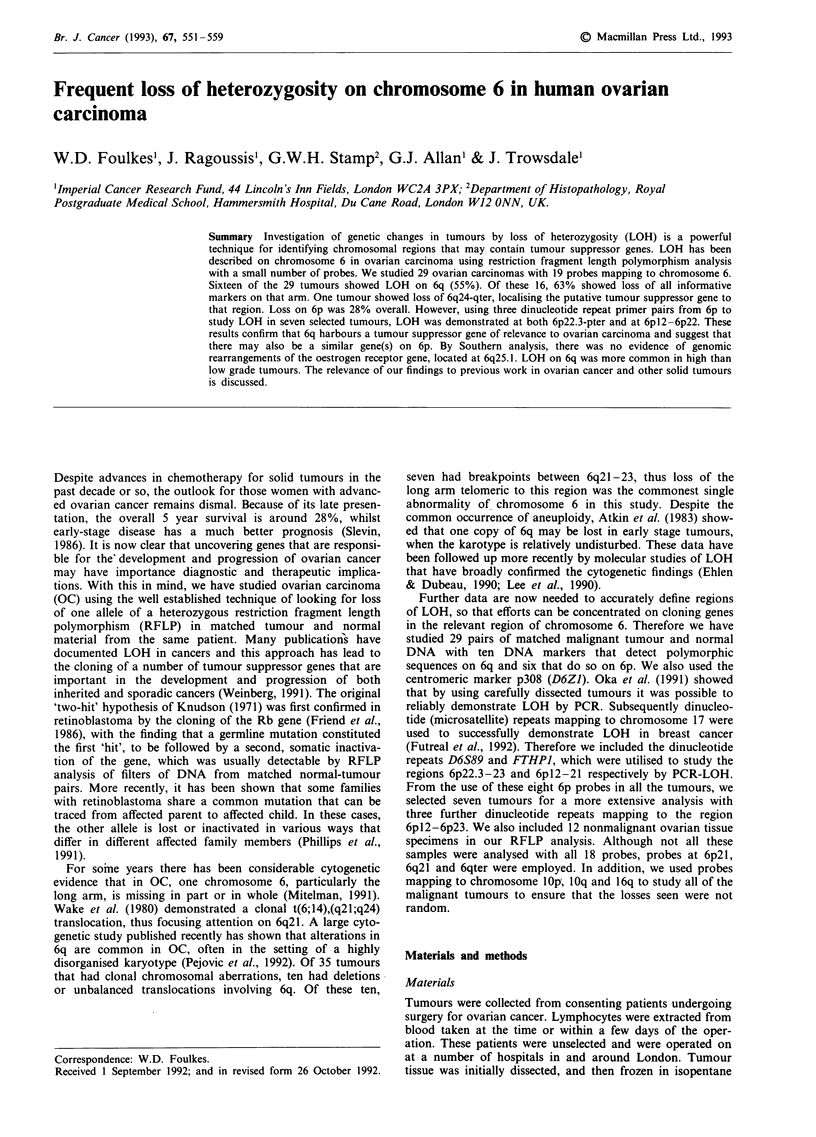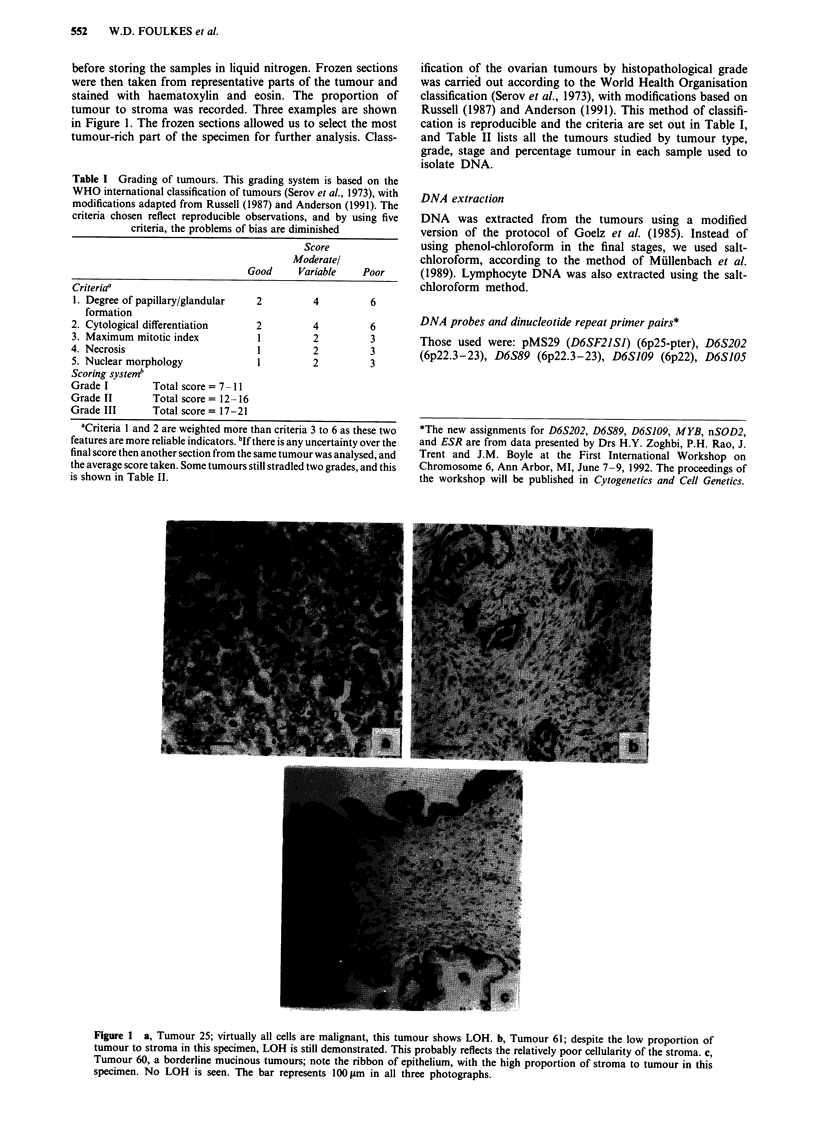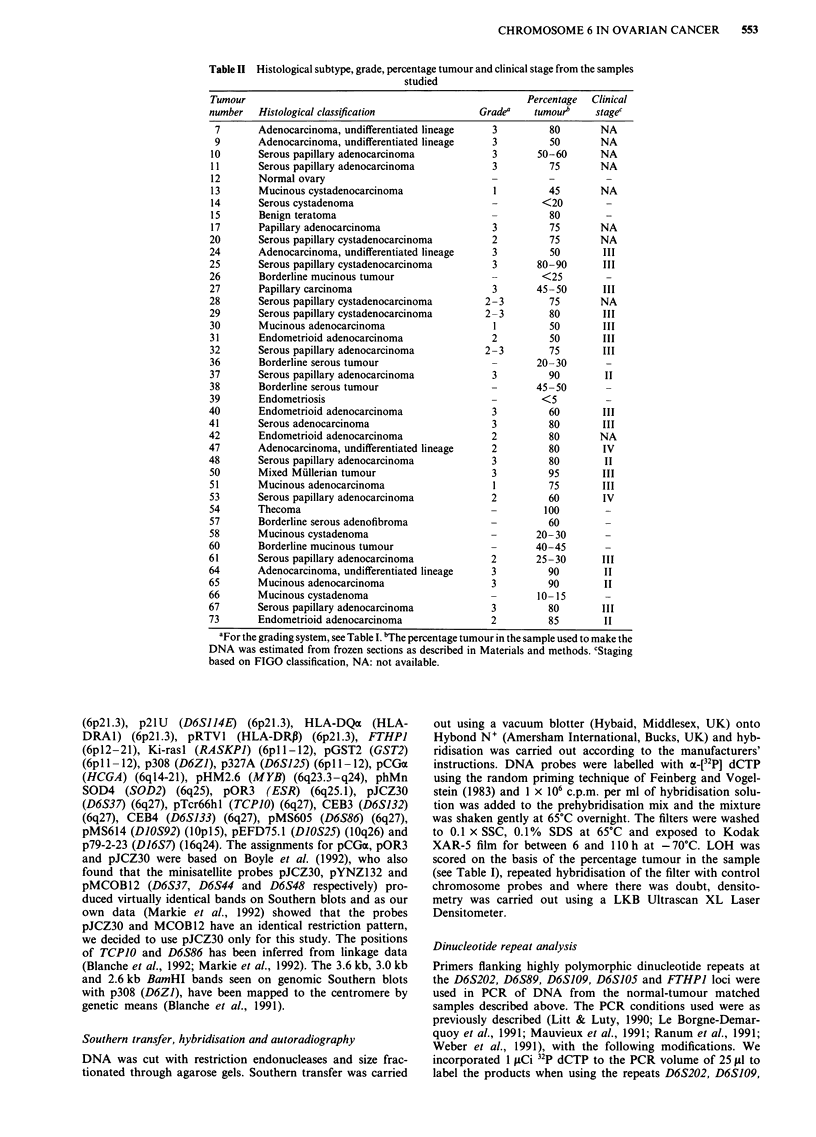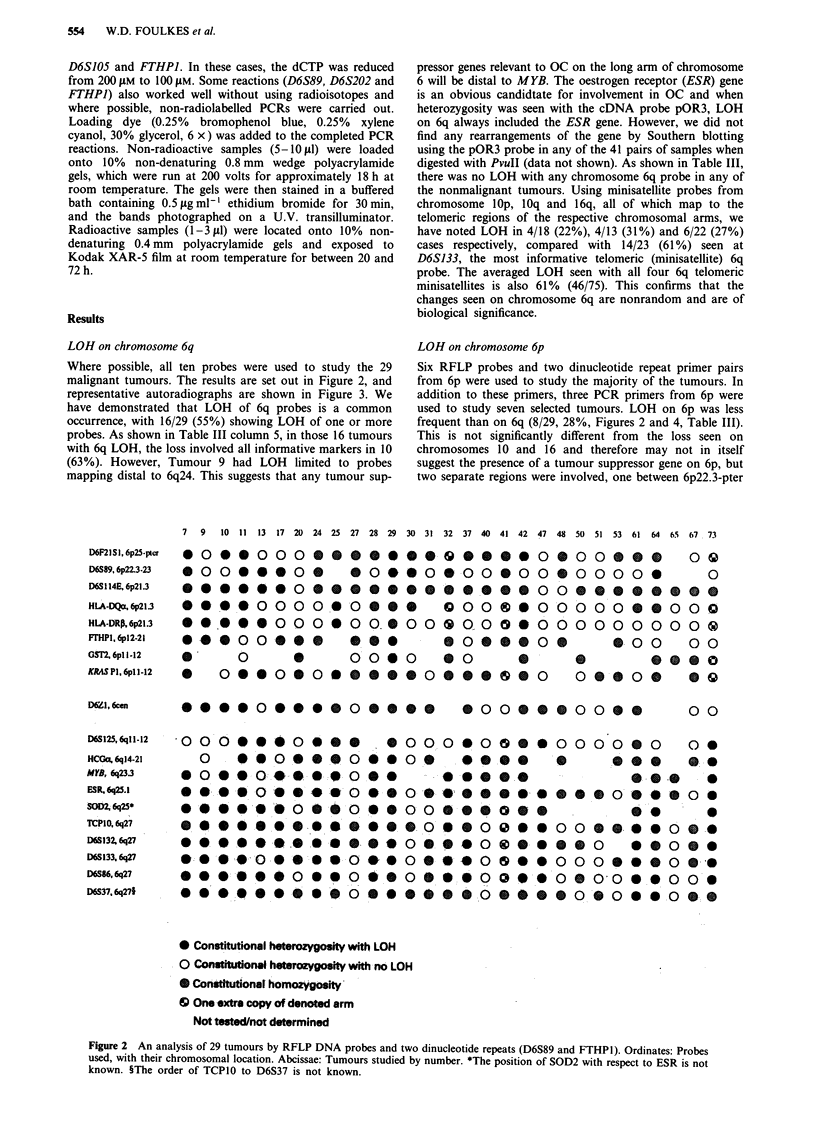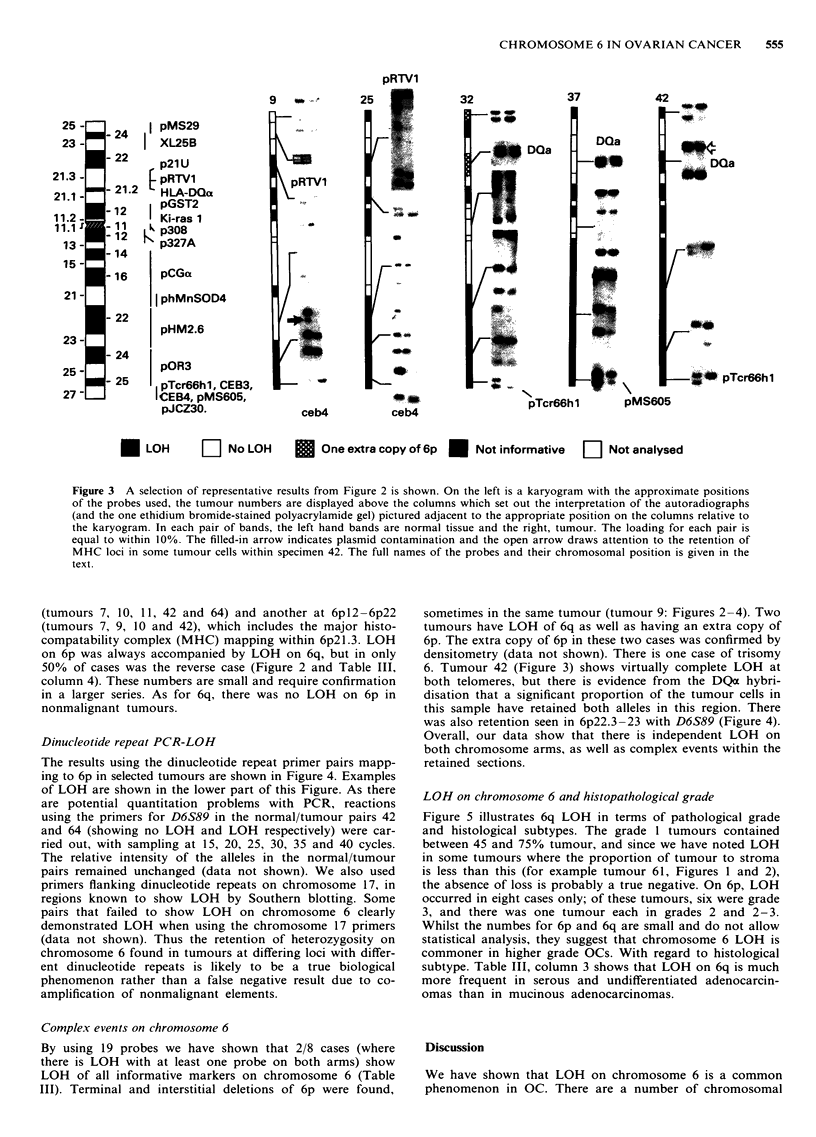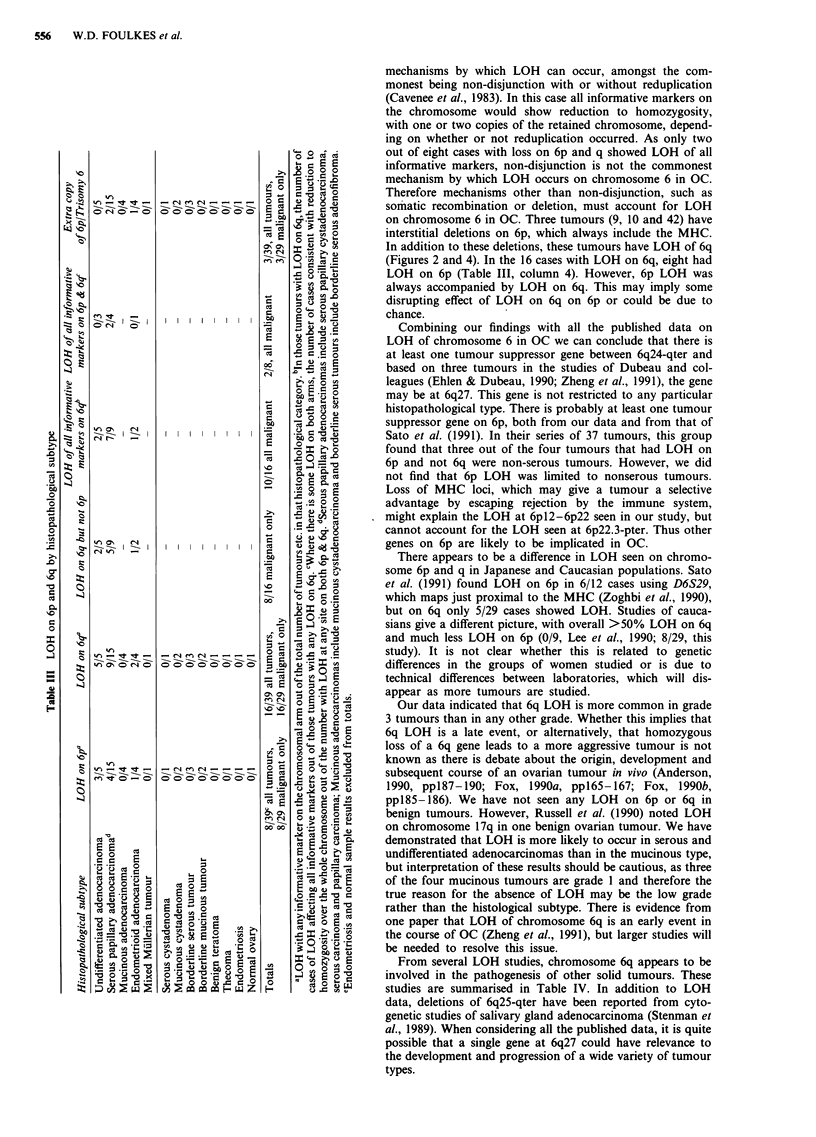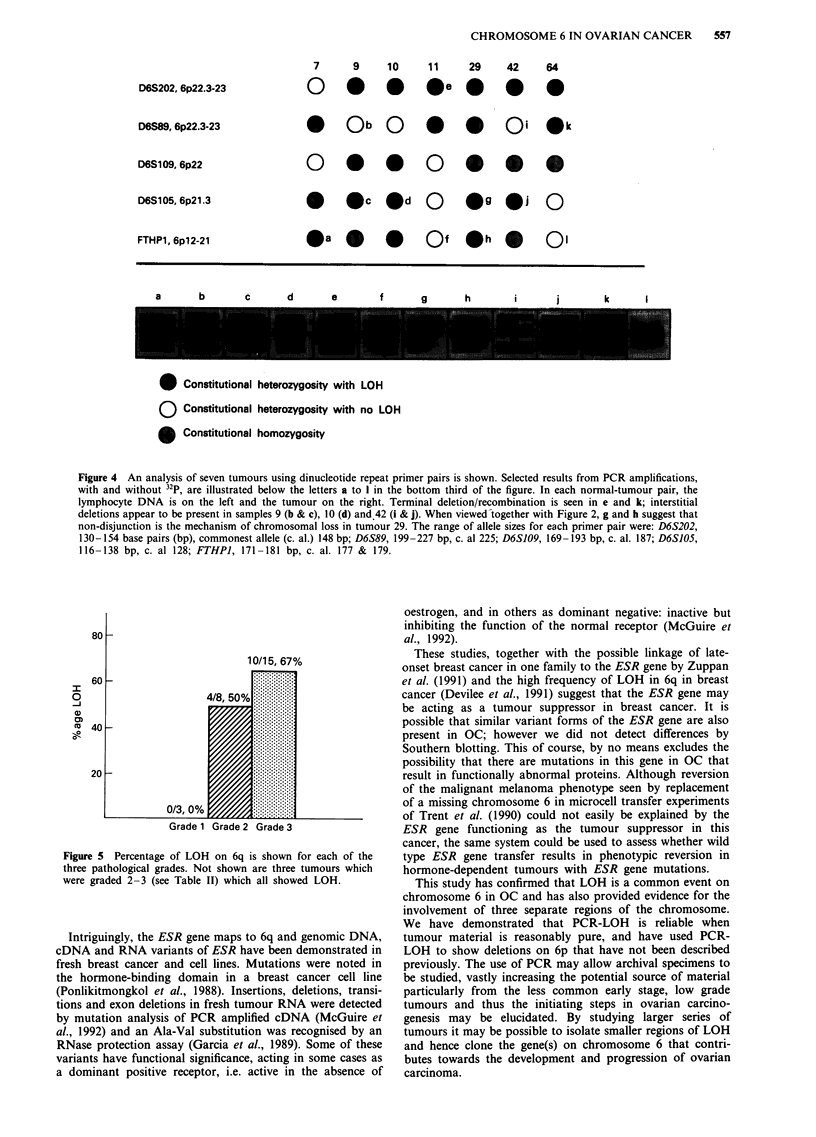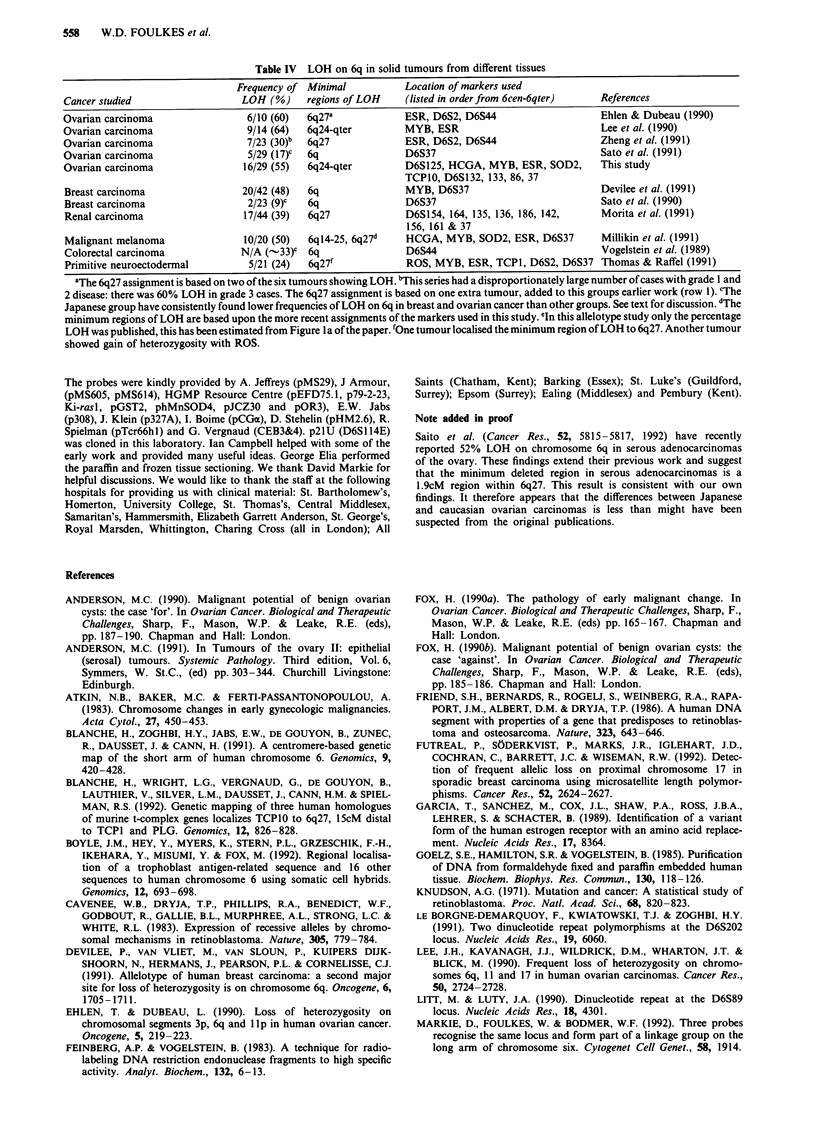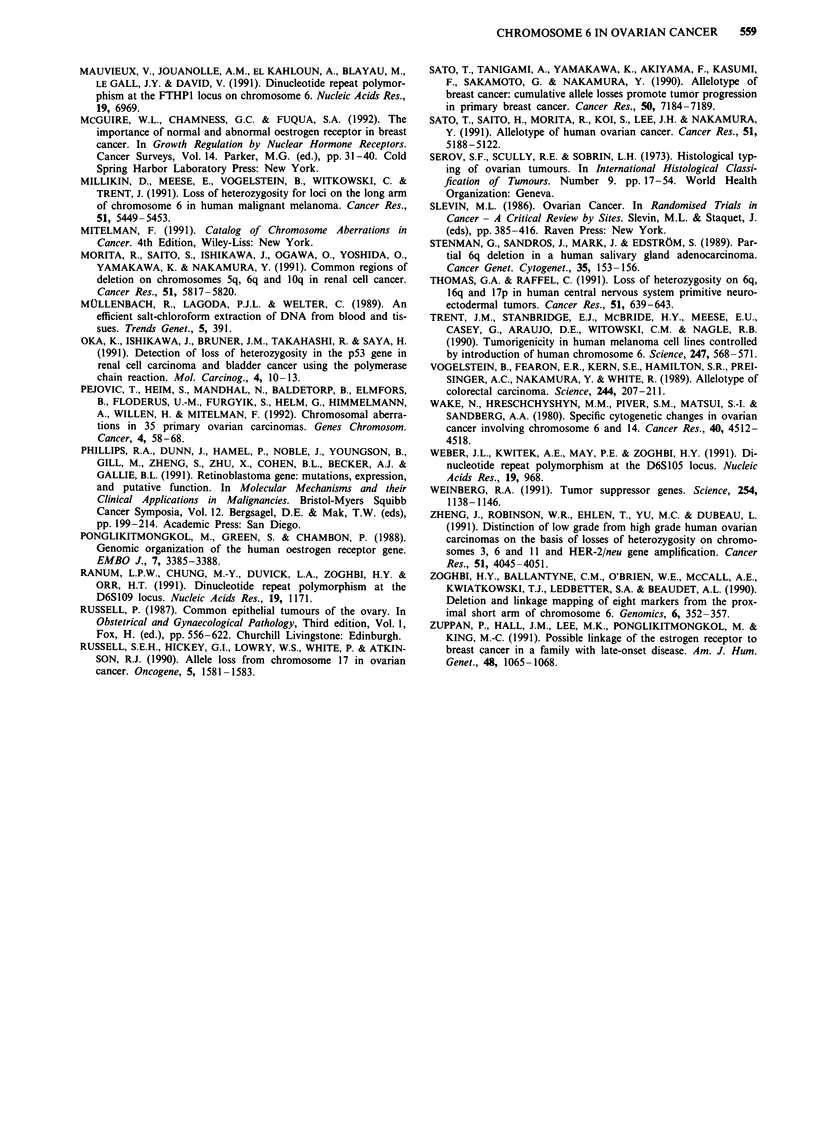# Frequent loss of heterozygosity on chromosome 6 in human ovarian carcinoma.

**DOI:** 10.1038/bjc.1993.101

**Published:** 1993-03

**Authors:** W. D. Foulkes, J. Ragoussis, G. W. Stamp, G. J. Allan, J. Trowsdale

**Affiliations:** Imperial Cancer Research Fund, Lincoln's Inn Fields, London, UK.

## Abstract

**Images:**


Br. J. Cancer (1993), 67, 551–559 © Macmillan Press Ltd., 1993

### Frequent loss of heterozygosity on chromosome 6 in human ovarian carcinoma

W.D. Foulkes[1], J. Ragoussis[1], G.W.H. Stamp[2], G.J. Allan[1] & J. Trowsdale[1]

[1]*Imperial Cancer Research Fund, 44 Lincoln's Inn Fields, London WC2A 3PX;* [2]*Department of Histopathology, Royal Postgraduate Medical School, Hammersmith Hospital, Du Cane Road, London W12 0NN, UK.*

**Summary** Investigation of genetic changes in tumours by loss of heterozygosity (LOH) is a powerful technique for identifying chromosomal regions that may contain tumour suppressor genes. LOH has been described on chromosome 6 in ovarian carcinoma using restriction fragment length polymorphism analysis with a small number of probes. We studied 29 ovarian carcinomas with 19 probes mapping to chromosome 6. Sixteen of the 29 tumours showed LOH on 6q (55%). Of these 16, 63% showed loss of all informative markers on that arm. One tumour showed loss of 6q24-qter, localising the putative tumour suppressor gene to that region. Loss on 6p was 28% overall. However, using three dinucleotide repeat primer pairs from 6p to study LOH in seven selected tumours, LOH was demonstrated at both 6p22.3-pter and at 6p12–6p22. These results confirm that 6q harbours a tumour suppressor gene of relevance to ovarian carcinoma and suggest that there may also be a similar gene(s) on 6p. By Southern analysis, there was no evidence of genomic rearrangements of the oestrogen receptor gene, located at 6q25.1. LOH on 6q was more common in high than low grade tumours. The relevance of our findings to previous work in ovarian cancer and other solid tumours is discussed.

Despite advances in chemotherapy for solid tumours in the past decade or so, the outlook for those women with advanced ovarian cancer remains dismal. Because of its late presentation, the overall 5 year survival is around 28%, whilst early-stage disease has a much better prognosis (Slevin, 1986). It is now clear that uncovering genes that are responsible for the development and progression of ovarian cancer may have importance diagnostic and therapeutic implications. With this in mind, we have studied ovarian carcinoma (OC) using the well established technique of looking for loss of one allele of a heterozygous restriction fragment length polymorphism (RFLP) in matched tumour and normal material from the same patient. Many publications have documented LOH in cancers and this approach has lead to the cloning of a number of tumour suppressor genes that are important in the development and progression of both inherited and sporadic cancers (Weinberg, 1991). The original 'two-hit' hypothesis of Knudson (1971) was first confirmed in retinoblastoma by the cloning of the Rb gene (Friend *et al.*, 1986), with the finding that a germline mutation constituted the first 'hit', to be followed by a second, somatic inactivation of the gene, which was usually detectable by RFLP analysis of filters of DNA from matched normal-tumour pairs. More recently, it has been shown that some families with retinoblastoma share a common mutation that can be traced from affected parent to affected child. In these cases, the other allele is lost or inactivated in various ways that differ in different affected family members (Phillips *et al.*, 1991).

For some years there has been considerable cytogenetic evidence that in OC, one chromosome 6, particularly the long arm, is missing in part or in whole (Mitelman, 1991). Wake *et al.* (1980) demonstrated a clonal t(6;14),(q21;q24) translocation, thus focusing attention on 6q21. A large cytogenetic study published recently has shown that alterations in 6q are common in OC, often in the setting of a highly disorganised karyotype (Pejovic *et al.*, 1992). Of 35 tumours that had clonal chromosomal aberrations, ten had deletions or unbalanced translocations involving 6q. Of these ten, seven had breakpoints between 6q21–23, thus loss of the long arm telomeric to this region was the commonest single abnormality of chromosome 6 in this study. Despite the common occurrence of aneuploidy, Atkin *et al.* (1983) showed that one copy of 6q may be lost in early stage tumours, when the karotype is relatively undisturbed. These data have been followed up more recently by molecular studies of LOH that have broadly confirmed the cytogenetic findings (Ehlen & Dubeau, 1990; Lee *et al.*, 1990).

Further data are now needed to accurately define regions of LOH, so that efforts can be concentrated on cloning genes in the relevant region of chromosome 6. Therefore we have studied 29 pairs of matched malignant tumour and normal DNA with ten DNA markers that detect polymorphic sequences on 6q and six that do so on 6p. We also used the centromeric marker p308 (*D6Z1*). Oka *et al.* (1991) showed that by using carefully dissected tumours it was possible to reliably demonstrate LOH by PCR. Subsequently dinucleotide (microsatellite) repeats mapping to chromosome 17 were used to successfully demonstrate LOH in breast cancer (Futreal *et al.*, 1992). Therefore we included the dinucleotide repeats *D6S89* and *FTHP1*, which were utilised to study the regions 6p22.3–23 and 6p12–21 respectively by PCR-LOH. From the use of these eight 6p probes in all the tumours, we selected seven tumours for a more extensive analysis with three further dinucleotide repeats mapping to the region 6p12–6p23. We also included 12 nonmalignant ovarian tissue specimens in our RFLP analysis. Although not all these samples were analysed with all 18 probes, probes at 6p21, 6q21 and 6qter were employed. In addition, we used probes mapping to chromosome 10p, 10q and 16q to study all of the malignant tumours to ensure that the losses seen were not random.

### Materials and methods

### *Materials*

Tumours were collected from consenting patients undergoing surgery for ovarian cancer. Lymphocytes were extracted from blood taken at the time or within a few days of the operation. These patients were unselected and were operated on at a number of hospitals in and around London. Tumour tissue was initially dissected, and then frozen in isopentane

Correspondence: W.D. Foulkes.
Received 1 September 1992; and in revised form 26 October 1992.

before storing the samples in liquid nitrogen. Frozen sections were then taken from representative parts of the tumour and stained with haematoxylin and eosin. The proportion of tumour to stroma was recorded. Three examples are shown in Figure 1. The frozen sections allowed us to select the most tumour-rich part of the specimen for further analysis. Classification of the ovarian tumours by histopathological grade was carried out according to the World Health Organisation classification (Serov *et al.*, 1973), with modifications based on Russell (1987) and Anderson (1991). This method of classification is reproducible and the criteria are set out in Table I, and Table II lists all the tumours studied by tumour type, grade, stage and percentage tumour in each sample used to isolate DNA.

**Table I** Grading of tumours. This grading system is based on the WHO international classification of tumours (Serov *et al.*, 1973), with modifications adapted from Russell (1987) and Anderson (1991). The criteria chosen reflect reproducible observations, and by using five criteria, the problems of bias are diminished

| | | *Score* | |
|---|---|---|---|
| | *Good* | *Moderate/ Variable* | *Poor* |
| *Criteria*[a] | | | |
| 1. Degree of papillary/glandular formation | 2 | 4 | 6 |
| 2. Cytological differentiation | 2 | 4 | 6 |
| 3. Maximum mitotic index | 1 | 2 | 3 |
| 4. Necrosis | 1 | 2 | 3 |
| 5. Nuclear morphology | 1 | 2 | 3 |
| *Scoring system*[b] | | | |
| Grade I — Total score = 7–11 | | | |
| Grade II — Total score = 12–16 | | | |
| Grade III — Total score = 17–21 | | | |

[a]Criteria 1 and 2 are weighted more than criteria 3 to 6 as these two features are more reliable indicators. [b]If there is any uncertainty over the final score then another section from the same tumour was analysed, and the average score taken. Some tumours still stradled two grades, and this is shown in Table II.

### *DNA extraction*

DNA was extracted from the tumours using a modified version of the protocol of Goelz *et al.* (1985). Instead of using phenol-chloroform in the final stages, we used salt-chloroform, according to the method of Müllenbach *et al.* (1989). Lymphocyte DNA was also extracted using the salt-chloroform method.

### *DNA probes and dinucleotide repeat primer pairs**

Those used were: pMS29 (*D6SF21S1*) (6p25-pter), *D6S202* (6p22.3–23), *D6S89* (6p22.3–23), *D6S109* (6p22), *D6S105*

*The new assignments for *D6S202, D6S89, D6S109, MYB*, n*SOD2*, and *ESR* are from data presented by Drs H.Y. Zoghbi, P.H. Rao, J. Trent and J.M. Boyle at the First International Workshop on Chromosome 6, Ann Arbor, MI, June 7–9, 1992. The proceedings of the workshop will be published in *Cytogenetics and Cell Genetics.*

**Figure 1** **a**, Tumour 25; virtually all cells are malignant, this tumour shows LOH. **b**, Tumour 61; despite the low proportion of tumour to stroma in this specimen, LOH is still demonstrated. This probably reflects the relatively poor cellularity of the stroma. **c**, Tumour 60, a borderline mucinous tumours; note the ribbon of epithelium, with the high proportion of stroma to tumour in this specimen. No LOH is seen. The bar represents 100 $\mu$m in all three photographs.

**Table II** Histological subtype, grade, percentage tumour and clinical stage from the samples studied

| *Tumour number* | *Histological classification* | *Grade*[a] | *Percentage tumour*[b] | *Clinical stage*[c] |
|---|---|---|---|---|
| 7 | Adenocarcinoma, undifferentiated lineage | 3 | 80 | NA |
| 9 | Adenocarcinoma, undifferentiated lineage | 3 | 50 | NA |
| 10 | Serous papillary adenocarcinoma | 3 | 50-60 | NA |
| 11 | Serous papillary adenocarcinoma | 3 | 75 | NA |
| 12 | Normal ovary | – | – | – |
| 13 | Mucinous cystadenocarcinoma | 1 | 45 | NA |
| 14 | Serous cystadenoma | – | <20 | – |
| 15 | Benign teratoma | – | 80 | – |
| 17 | Papillary adenocarcinoma | 3 | 75 | NA |
| 20 | Serous papillary cystadenocarcinoma | 2 | 75 | NA |
| 24 | Adenocarcinoma, undifferentiated lineage | 3 | 50 | III |
| 25 | Serous papillary cystadenocarcinoma | 3 | 80-90 | III |
| 26 | Borderline mucinous tumour | – | <25 | – |
| 27 | Papillary carcinoma | 3 | 45-50 | III |
| 28 | Serous papillary cystadenocarcinoma | 2-3 | 75 | NA |
| 29 | Serous papillary cystadenocarcinoma | 2-3 | 80 | III |
| 30 | Mucinous adenocarcinoma | 1 | 50 | III |
| 31 | Endometrioid adenocarcinoma | 2 | 50 | III |
| 32 | Serous papillary adenocarcinoma | 2-3 | 75 | III |
| 36 | Borderline serous tumour | – | 20-30 | – |
| 37 | Serous papillary adenocarcinoma | 3 | 90 | II |
| 38 | Borderline serous tumour | – | 45-50 | – |
| 39 | Endometriosis | – | <5 | – |
| 40 | Endometrioid adenocarcinoma | 3 | 60 | III |
| 41 | Serous adenocarcinoma | 3 | 80 | III |
| 42 | Endometrioid adenocarcinoma | 2 | 80 | NA |
| 47 | Adenocarcinoma, undifferentiated lineage | 2 | 80 | IV |
| 48 | Serous papillary adenocarcinoma | 3 | 80 | II |
| 50 | Mixed Müllerian tumour | 3 | 95 | III |
| 51 | Mucinous adenocarcinoma | 1 | 75 | III |
| 53 | Serous papillary adenocarcinoma | 2 | 60 | IV |
| 54 | Thecoma | – | 100 | – |
| 57 | Borderline serous adenofibroma | – | 60 | – |
| 58 | Mucinous cystadenoma | – | 20-30 | – |
| 60 | Borderline mucinous tumour | – | 40-45 | – |
| 61 | Serous papillary adenocarcinoma | 2 | 25-30 | III |
| 64 | Adenocarcinoma, undifferentiated lineage | 3 | 90 | II |
| 65 | Mucinous adenocarcinoma | 3 | 90 | II |
| 66 | Mucinous cystadenoma | – | 10-15 | – |
| 67 | Serous papillary adenocarcinoma | 3 | 80 | III |
| 73 | Endometrioid adenocarcinoma | 2 | 85 | II |

[a]For the grading system, see Table I. [b]The percentage tumour in the sample used to make the DNA was estimated from frozen sections as described in Materials and methods. [c]Staging based on FIGO classification, NA: not available.

(6p21.3), p21U (*D6S114E*) (6p21.3), HLA-DQα (HLA-DRA1) (6p21.3), pRTV1 (HLA-DRβ) (6p21.3), *FTHP1* (6p12-21), Ki-ras1 (*RASKP1*) (6p11-12), pGST2 (*GST2*) (6p11-12), p308 (*D6Z1*), p327A (*D6S125*) (6p11-12), pCGα (*HCGA*) (6q14-21), pHM2.6 (*MYB*) (6q23.3-q24), phMn SOD4 (*SOD2*) (6q25), pOR3 (*ESR*) (6q25.1), pJCZ30 (*D6S37*) (6q27), pTcr66h1 (*TCP10*) (6q27), CEB3 (*D6S132*) (6q27), CEB4 (*D6S133*) (6q27), pMS605 (*D6S86*) (6q27), pMS614 (*D10S92*) (10p15), pEFD75.1 (*D10S25*) (10q26) and p79-2-23 (*D16S7*) (16q24). The assignments for pCGα, pOR3 and pJCZ30 were based on Boyle *et al.* (1992), who also found that the minisatellite probes pJCZ30, pYNZ132 and pMCOB12 (*D6S37*, *D6S44* and *D6S48* respectively) produced virtually identical bands on Southern blots and as our own data (Markie *et al.*, 1992) showed that the probes pJCZ30 and MCOB12 have an identical restriction pattern, we decided to use pJCZ30 only for this study. The positions of *TCP10* and *D6S86* has been inferred from linkage data (Blanche *et al.*, 1992; Markie *et al.*, 1992). The 3.6 kb, 3.0 kb and 2.6 kb *Bam*HI bands seen on genomic Southern blots with p308 (*D6Z1*), have been mapped to the centromere by genetic means (Blanche *et al.*, 1991).

### *Southern transfer, hybridisation and autoradiography*

DNA was cut with restriction endonucleases and size fractionated through agarose gels. Southern transfer was carried out using a vacuum blotter (Hybaid, Middlesex, UK) onto Hybond N$^+$ (Amersham International, Bucks, UK) and hybridisation was carried out according to the manufacturers' instructions. DNA probes were labelled with α-[$^{32}$P] dCTP using the random priming technique of Feinberg and Vogelstein (1983) and 1 × 10$^6$ c.p.m. per ml of hybridisation solution was added to the prehybridisation mix and the mixture was shaken gently at 65°C overnight. The filters were washed to 0.1 × SSC, 0.1% SDS at 65°C and exposed to Kodak XAR-5 film for between 6 and 110 h at −70°C. LOH was scored on the basis of the percentage tumour in the sample (see Table I), repeated hybridisation of the filter with control chromosome probes and where there was doubt, densitometry was carried out using a LKB Ultrascan XL Laser Densitometer.

### *Dinucleotide repeat analysis*

Primers flanking highly polymorphic dinucleotide repeats at the *D6S202, D6S89, D6S109, D6S105* and *FTHP1* loci were used in PCR of DNA from the normal-tumour matched samples described above. The PCR conditions used were as previously described (Litt & Luty, 1990; Le Borgne-Demarquoy *et al.*, 1991; Mauvieux *et al.*, 1991; Ranum *et al.*, 1991; Weber *et al.*, 1991), with the following modifications. We incorporated 1 μCi $^{32}$P dCTP to the PCR volume of 25 μl to label the products when using the repeats *D6S202, D6S109,*

*D6S105* and *FTHP1*. In these cases, the dCTP was reduced from 200 μM to 100 μM. Some reactions (*D6S89, D6S202* and *FTHP1*) also worked well without using radioisotopes and where possible, non-radiolabelled PCRs were carried out. Loading dye (0.25% bromophenol blue, 0.25% xylene cyanol, 30% glycerol, 6 ×) was added to the completed PCR reactions. Non-radioactive samples (5–10 μl) were loaded onto 10% non-denaturing 0.8 mm wedge polyacrylamide gels, which were run at 200 volts for approximately 18 h at room temperature. The gels were then stained in a buffered bath containing 0.5 $\mu g\ ml^{-1}$ ethidium bromide for 30 min, and the bands photographed on a U.V. transilluminator. Radioactive samples (1–3 μl) were located onto 10% non-denaturing 0.4 mm polyacrylamide gels and exposed to Kodak XAR-5 film at room temperature for between 20 and 72 h.

### Results

### *LOH on chromosome 6q*

Where possible, all ten probes were used to study the 29 malignant tumours. The results are set out in Figure 2, and representative autoradiographs are shown in Figure 3. We have demonstrated that LOH of 6q probes is a common occurrence, with 16/29 (55%) showing LOH of one or more probes. As shown in Table III column 5, in those 16 tumours with 6q LOH, the loss involved all informative markers in 10 (63%). However, Tumour 9 had LOH limited to probes mapping distal to 6q24. This suggests that any tumour suppressor genes relevant to OC on the long arm of chromosome 6 will be distal to *MYB*. The oestrogen receptor (*ESR*) gene is an obvious candidtate for involvement in OC and when heterozygosity was seen with the cDNA probe pOR3, LOH on 6q always included the *ESR* gene. However, we did not find any rearrangements of the gene by Southern blotting using the pOR3 probe in any of the 41 pairs of samples when digested with *Pvu*II (data not shown). As shown in Table III, there was no LOH with any chromosome 6q probe in any of the nonmalignant tumours. Using minisatellite probes from chromosome 10p, 10q and 16q, all of which map to the telomeric regions of the respective chromosomal arms, we have noted LOH in 4/18 (22%), 4/13 (31%) and 6/22 (27%) cases respectively, compared with 14/23 (61%) seen at *D6S133*, the most informative telomeric (minisatellite) 6q probe. The averaged LOH seen with all four 6q telomeric minisatellites is also 61% (46/75). This confirms that the changes seen on chromosome 6q are nonrandom and are of biological significance.

### *LOH on chromosome 6p*

Six RFLP probes and two dinucleotide repeat primer pairs from 6p were used to study the majority of the tumours. In addition to these primers, three PCR primers from 6p were used to study seven selected tumours. LOH on 6p was less frequent than on 6q (8/29, 28%, Figures 2 and 4, Table III). This is not significantly different from the loss seen on chromosomes 10 and 16 and therefore may not in itself suggest the presence of a tumour suppressor gene on 6p, but two separate regions were involved, one between 6p22.3-pter

**Figure 2** An analysis of 29 tumours by RFLP DNA probes and two dinucleotide repeats (D6S89 and FTHP1). Ordinates: Probes used, with their chromosomal location. Abcissae: Tumours studied by number. *The position of SOD2 with respect to ESR is not known. §The order of TCP10 to D6S37 is not known.

**Figure 3** A selection of representative results from Figure 2 is shown. On the left is a karyogram with the approximate positions of the probes used, the tumour numbers are displayed above the columns which set out the interpretation of the autoradiographs (and the one ethidium bromide-stained polyacrylamide gel) pictured adjacent to the appropriate position on the columns relative to the karyogram. In each pair of bands, the left hand bands are normal tissue and the right, tumour. The loading for each pair is equal to within 10%. The filled-in arrow indicates plasmid contamination and the open arrow draws attention to the retention of MHC loci in some tumour cells within specimen 42. The full names of the probes and their chromosomal position is given in the text.

(tumours 7, 10, 11, 42 and 64) and another at 6p12–6p22 (tumours 7, 9, 10 and 42), which includes the major histocompatability complex (MHC) mapping within 6p21.3. LOH on 6p was always accompanied by LOH on 6q, but in only 50% of cases was the reverse case (Figure 2 and Table III, column 4). These numbers are small and require confirmation in a larger series. As for 6q, there was no LOH on 6p in nonmalignant tumours.

*Dinucleotide repeat PCR-LOH*

The results using the dinucleotide repeat primer pairs mapping to 6p in selected tumours are shown in Figure 4. Examples of LOH are shown in the lower part of this Figure. As there are potential quantitation problems with PCR, reactions using the primers for *D6S89* in the normal/tumour pairs 42 and 64 (showing no LOH and LOH respectively) were carried out, with sampling at 15, 20, 25, 30, 35 and 40 cycles. The relative intensity of the alleles in the normal/tumour pairs remained unchanged (data not shown). We also used primers flanking dinucleotide repeats on chromosome 17, in regions known to show LOH by Southern blotting. Some pairs that failed to show LOH on chromosome 6 clearly demonstrated LOH when using the chromosome 17 primers (data not shown). Thus the retention of heterozygosity on chromosome 6 found in tumours at differing loci with different dinucleotide repeats is likely to be a true biological phenomenon rather than a false negative result due to co-amplification of nonmalignant elements.

*Complex events on chromosome 6*

By using 19 probes we have shown that 2/8 cases (where there is LOH with at least one probe on both arms) show LOH of all informative markers on chromosome 6 (Table III). Terminal and interstitial deletions of 6p were found, sometimes in the same tumour (tumour 9: Figures 2–4). Two tumours have LOH of 6q as well as having an extra copy of 6p. The extra copy of 6p in these two cases was confirmed by densitometry (data not shown). There is one case of trisomy 6. Tumour 42 (Figure 3) shows virtually complete LOH at both telomeres, but there is evidence from the DQα hybridisation that a significant proportion of the tumour cells in this sample have retained both alleles in this region. There was also retention seen in 6p22.3–23 with *D6S89* (Figure 4). Overall, our data show that there is independent LOH on both chromosome arms, as well as complex events within the retained sections.

*LOH on chromosome 6 and histopathological grade*

Figure 5 illustrates 6q LOH in terms of pathological grade and histological subtypes. The grade 1 tumours contained between 45 and 75% tumour, and since we have noted LOH in some tumours where the proportion of tumour to stroma is less than this (for example tumour 61, Figures 1 and 2), the absence of loss is probably a true negative. On 6p, LOH occurred in eight cases only; of these tumours, six were grade 3, and there was one tumour each in grades 2 and 2–3. Whilst the numbes for 6p and 6q are small and do not allow statistical analysis, they suggest that chromosome 6 LOH is commoner in higher grade OCs. With regard to histological subtype. Table III, column 3 shows that LOH on 6q is much more frequent in serous and undifferentiated adenocarcinomas than in mucinous adenocarcinomas.

### Discussion

We have shown that LOH on chromosome 6 is a common phenomenon in OC. There are a number of chromosomal

**Table III** LOH on 6p and 6q by histopathological subtype

| *Histopathological subtype* | *LOH on 6p*[a] | *LOH on 6q*[a] | *LOH on 6q but not 6p* | *LOH of all informative markers on 6q*[b] | *LOH of all informative markers on 6p & 6q*[c] | *Extra copy of 6p/Trisomy 6* |
|---|---|---|---|---|---|---|
| Undifferentiated adenocarcinoma | 3/5 | 5/5 | 2/5 | 2/5 | 0/3 | 0/5 |
| Serous papillary adenocarcinoma[d] | 4/15 | 9/15 | 5/9 | 7/9 | 2/4 | 2/15 |
| Mucinous adenocarcinoma | 0/4 | 0/4 | – | – | – | 0/4 |
| Endometrioid adenocarcinoma | 1/4 | 2/4 | 1/2 | 1/2 | 0/1 | 1/4 |
| Mixed Müllerian tumour | 0/1 | 0/1 | – | – | – | 0/1 |
| Serous cystadenoma | 0/1 | 0/1 | – | – | – | 0/1 |
| Mucinous cystadenoma | 0/2 | 0/2 | – | – | – | 0/2 |
| Borderline serous tumour | 0/3 | 0/3 | – | – | – | 0/3 |
| Borderline mucinous tumour | 0/2 | 0/2 | – | – | – | 0/2 |
| Benign teratoma | 0/1 | 0/1 | – | – | – | 0/1 |
| Thecoma | 0/1 | 0/1 | – | – | – | 0/1 |
| Endometriosis | 0/1 | 0/1 | – | – | – | 0/1 |
| Normal ovary | 0/1 | 0/1 | – | – | – | 0/1 |
| Totals | 8/39[e] all tumours, 8/29 malignant only | 16/39 all tumours, 16/29 malignant only | 8/16 malignant only | 10/16 all malignant | 2/8, all malignant | 3/39, all tumours, 3/29 malignant only |

[a]LOH with any informative marker on the chromosomal arm out of the total number of tumours etc. in that histopathological category. [b]In those tumours with LOH on 6q, the number of cases of LOH affecting all informative markers out of those tumours with any LOH on 6q. [c]Where there is some LOH on both arms, the number of cases consistent with reduction to homozygosity over the whole chromosome out of the number with LOH at any site on both 6p & 6q. [d]Serous papillary adenocarcinomas include serous papillary cystadenocarcinoma, serous carcinoma and papillary carcinoma; Mucinous adenocarcinomas include mucinous cystadenocarcinoma and borderline serous tumours include borderline serous adenofibroma. [e]Endometriosis and normal sample results excluded from totals.

mechanisms by which LOH can occur, amongst the commonest being non-disjunction with or without reduplication (Cavenee *et al.*, 1983). In this case all informative markers on the chromosome would show reduction to homozygosity, with one or two copies of the retained chromosome, depending on whether or not reduplication occurred. As only two out of eight cases with loss on 6p and q showed LOH of all informative markers, non-disjunction is not the commonest mechanism by which LOH occurs on chromosome 6 in OC. Therefore mechanisms other than non-disjunction, such as somatic recombination or deletion, must account for LOH on chromosome 6 in OC. Three tumours (9, 10 and 42) have interstitial deletions on 6p, which always include the MHC. In addition to these deletions, these tumours have LOH of 6q (Figures 2 and 4). In the 16 cases with LOH on 6q, eight had LOH on 6p (Table III, column 4). However, 6p LOH was always accompanied by LOH on 6q. This may imply some disrupting effect of LOH on 6q on 6p or could be due to chance.

Combining our findings with all the published data on LOH of chromosome 6 in OC we can conclude that there is at least one tumour suppressor gene between 6q24-qter and based on three tumours in the studies of Dubeau and colleagues (Ehlen & Dubeau, 1990; Zheng *et al.*, 1991), the gene may be at 6q27. This gene is not restricted to any particular histopathological type. There is probably at least one tumour suppressor gene on 6p, both from our data and from that of Sato *et al.* (1991). In their series of 37 tumours, this group found that three out of the four tumours that had LOH on 6p and not 6q were non-serous tumours. However, we did not find that 6p LOH was limited to nonserous tumours. Loss of MHC loci, which may give a tumour a selective advantage by escaping rejection by the immune system, might explain the LOH at 6p12–6p22 seen in our study, but cannot account for the LOH seen at 6p22.3-pter. Thus other genes on 6p are likely to be implicated in OC.

There appears to be a difference in LOH seen on chromosome 6p and q in Japanese and Caucasian populations. Sato *et al.* (1991) found LOH on 6p in 6/12 cases using *D6S29*, which maps just proximal to the MHC (Zoghbi *et al.*, 1990), but on 6q only 5/29 cases showed LOH. Studies of caucasians give a different picture, with overall >50% LOH on 6q and much less LOH on 6p (0/9, Lee *et al.*, 1990; 8/29, this study). It is not clear whether this is related to genetic differences in the groups of women studied or is due to technical differences between laboratories, which will disappear as more tumours are studied.

Our data indicated that 6q LOH is more common in grade 3 tumours than in any other grade. Whether this implies that 6q LOH is a late event, or alternatively, that homozygous loss of a 6q gene leads to a more aggressive tumour is not known as there is debate about the origin, development and subsequent course of an ovarian tumour *in vivo* (Anderson, 1990, pp187–190; Fox, 1990*a*, pp165–167; Fox, 1990*b*, pp185–186). We have not seen any LOH on 6p or 6q in benign tumours. However, Russell *et al.* (1990) noted LOH on chromosome 17q in one benign ovarian tumour. We have demonstrated that LOH is more likely to occur in serous and undifferentiated adenocarcinomas than in the mucinous type, but interpretation of these results should be cautious, as three of the four mucinous tumours are grade 1 and therefore the true reason for the absence of LOH may be the low grade rather than the histological subtype. There is evidence from one paper that LOH of chromosome 6q is an early event in the course of OC (Zheng *et al.*, 1991), but larger studies will be needed to resolve this issue.

From several LOH studies, chromosome 6q appears to be involved in the pathogenesis of other solid tumours. These studies are summarised in Table IV. In addition to LOH data, deletions of 6q25-qter have been reported from cytogenetic studies of salivary gland adenocarcinoma (Stenman *et al.*, 1989). When considering all the published data, it is quite possible that a single gene at 6q27 could have relevance to the development and progression of a wide variety of tumour types.

**Figure 4** An analysis of seven tumours using dinucleotide repeat primer pairs is shown. Selected results from PCR amplifications, with and without $^{32}P$, are illustrated below the letters **a** to **l** in the bottom third of the figure. In each normal-tumour pair, the lymphocyte DNA is on the left and the tumour on the right. Terminal deletion/recombination is seen in **e** and **k**; interstitial deletions appear to be present in samples 9 (**b** & **c**), 10 (**d**) and 42 (**i** & **j**). When viewed together with Figure 2, **g** and **h** suggest that non-disjunction is the mechanism of chromosomal loss in tumour 29. The range of allele sizes for each primer pair were: *D6S202*, 130–154 base pairs (bp), commonest allele (c. al.) 148 bp; *D6S89*, 199–227 bp, c. al 225; *D6S109*, 169–193 bp, c. al. 187; *D6S105*, 116–138 bp, c. al 128; *FTHP1*, 171–181 bp, c. al. 177 & 179.

**Figure 5** Percentage of LOH on 6q is shown for each of the three pathological grades. Not shown are three tumours which were graded 2–3 (see Table II) which all showed LOH.

Intriguingly, the *ESR* gene maps to 6q and genomic DNA, cDNA and RNA variants of *ESR* have been demonstrated in fresh breast cancer and cell lines. Mutations were noted in the hormone-binding domain in a breast cancer cell line (Ponlikitmongkol *et al.*, 1988). Insertions, deletions, transitions and exon deletions in fresh tumour RNA were detected by mutation analysis of PCR amplified cDNA (McGuire *et al.*, 1992) and an Ala-Val substitution was recognised by an RNase protection assay (Garcia *et al.*, 1989). Some of these variants have functional significance, acting in some cases as a dominant positive receptor, i.e. active in the absence of oestrogen, and in others as dominant negative: inactive but inhibiting the function of the normal receptor (McGuire *et al.*, 1992).

These studies, together with the possible linkage of late-onset breast cancer in one family to the *ESR* gene by Zuppan *et al.* (1991) and the high frequency of LOH in 6q in breast cancer (Devilee *et al.*, 1991) suggest that the *ESR* gene may be acting as a tumour suppressor in breast cancer. It is possible that similar variant forms of the *ESR* gene are also present in OC; however we did not detect differences by Southern blotting. This of course, by no means excludes the possibility that there are mutations in this gene in OC that result in functionally abnormal proteins. Although reversion of the malignant melanoma phenotype seen by replacement of a missing chromosome 6 in microcell transfer experiments of Trent *et al.* (1990) could not easily be explained by the *ESR* gene functioning as the tumour suppressor in this cancer, the same system could be used to assess whether wild type *ESR* gene transfer results in phenotypic reversion in hormone-dependent tumours with *ESR* gene mutations.

This study has confirmed that LOH is a common event on chromosome 6 in OC and has also provided evidence for the involvement of three separate regions of the chromosome. We have demonstrated that PCR-LOH is reliable when tumour material is reasonably pure, and have used PCR-LOH to show deletions on 6p that have not been described previously. The use of PCR may allow archival specimens to be studied, vastly increasing the potential source of material particularly from the less common early stage, low grade tumours and thus the initiating steps in ovarian carcinogenesis may be elucidated. By studying larger series of tumours it may be possible to isolate smaller regions of LOH and hence clone the gene(s) on chromosome 6 that contributes towards the development and progression of ovarian carcinoma.

**Table IV** LOH on 6q in solid tumours from different tissues

| Cancer studied | Frequency of LOH (%) | Minimal regions of LOH | Location of markers used (listed in order from 6cen-6qter) | References |
|---|---|---|---|---|
| Ovarian carcinoma | 6/10 (60) | 6q27[a] | ESR, D6S2, D6S44 | Ehlen & Dubeau (1990) |
| Ovarian carcinoma | 9/14 (64) | 6q24-qter | MYB, ESR | Lee *et al.* (1990) |
| Ovarian carcinoma | 7/23 (30)[b] | 6q27 | ESR, D6S2, D6S44 | Zheng *et al.* (1991) |
| Ovarian carcinoma | 5/29 (17)[c] | 6q | D6S37 | Sato *et al.* (1991) |
| Ovarian carcinoma | 16/29 (55) | 6q24-qter | D6S125, HCGA, MYB, ESR, SOD2, TCP10, D6S132, 133, 86, 37 | This study |
| Breast carcinoma | 20/42 (48) | 6q | MYB, D6S37 | Devilee *et al.* (1991) |
| Breast carcinoma | 2/23 (9)[c] | 6q | D6S37 | Sato *et al.* (1990) |
| Renal carcinoma | 17/44 (39) | 6q27 | D6S154, 164, 135, 136, 186, 142, 156, 161 & 37 | Morita *et al.* (1991) |
| Malignant melanoma | 10/20 (50) | 6q14-25, 6q27[d] | HCGA, MYB, SOD2, ESR, D6S37 | Millikin *et al.* (1991) |
| Colorectal carcinoma | N/A (~33)[e] | 6q | D6S44 | Vogelstein *et al.* (1989) |
| Primitive neuroectodermal | 5/21 (24) | 6q27[f] | ROS, MYB, ESR, TCP1, D6S2, D6S37 | Thomas & Raffel (1991) |

[a]The 6q27 assignment is based on two of the six tumours showing LOH. [b]This series had a disproportionately large number of cases with grade 1 and 2 disease: there was 60% LOH in grade 3 cases. The 6q27 assignment is based on one extra tumour, added to this groups earlier work (row 1). [c]The Japanese group have consistently found lower frequencies of LOH on 6q in breast and ovarian cancer than other groups. See text for discussion. [d]The minimum regions of LOH are based upon the more recent assignments of the markers used in this study. [e]In this allelotype study only the percentage LOH was published, this has been estimated from Figure 1a of the paper. [f]One tumour localised the minimum region of LOH to 6q27. Another tumour showed gain of heterozygosity with ROS.

The probes were kindly provided by A. Jeffreys (pMS29), J Armour, (pMS605, pMS614), HGMP Resource Centre (pEFD75.1, p79-2-23, Ki-*ras*1, pGST2, phMnSOD4, pJCZ30 and pOR3), E.W. Jabs (p308), J. Klein (p327A), I. Boime (pCGα), D. Stehelin (pHM2.6), R. Spielman (pTcr66h1) and G. Vergnaud (CEB3&4). p21U (D6S114E) was cloned in this laboratory. Ian Campbell helped with some of the early work and provided many useful ideas. George Elia performed the paraffin and frozen tissue sectioning. We thank David Markie for helpful discussions. We would like to thank the staff at the following hospitals for providing us with clinical material: St. Bartholomew's, Homerton, University College, St. Thomas's, Central Middlesex, Samaritan's, Hammersmith, Elizabeth Garrett Anderson, St. George's, Royal Marsden, Whittington, Charing Cross (all in London); All Saints (Chatham, Kent); Barking (Essex); St. Luke's (Guildford, Surrey); Epsom (Surrey); Ealing (Middlesex) and Pembury (Kent).

**Note added in proof**

Saito *et al.* (*Cancer Res.*, **52**, 5815-5817, 1992) have recently reported 52% LOH on chromosome 6q in serous adenocarcinomas of the ovary. These findings extend their previous work and suggest that the minimum deleted region in serous adenocarcinomas is a 1.9cM region within 6q27. This result is consistent with our own findings. It therefore appears that the differences between Japanese and caucasian ovarian carcinomas is less than might have been suspected from the original publications.

### References

ANDERSON, M.C. (1990). Malignant potential of benign ovarian cysts: the case 'for'. In *Ovarian Cancer. Biological and Therapeutic Challenges*, Sharp, F., Mason, W.P. & Leake, R.E. (eds), pp. 187-190. Chapman and Hall: London.

ANDERSON, M.C. (1991). In Tumours of the ovary II: epithelial (serosal) tumours. *Systemic Pathology*. Third edition, Vol. 6, Symmers, W. St.C., (ed) pp. 303-344. Churchill Livingstone: Edinburgh.

ATKIN, N.B., BAKER, M.C. & FERTI-PASSANTONOPOULOU, A. (1983). Chromosome changes in early gynecologic malignancies. *Acta Cytol.*, **27**, 450-453.

BLANCHE, H., ZOGHBI, H.Y., JABS, E.W., DE GOUYON, B., ZUNEC, R., DAUSSET, J. & CANN, H. (1991). A centromere-based genetic map of the short arm of human chromosome 6. *Genomics*, **9**, 420-428.

BLANCHE, H., WRIGHT, L.G., VERGNAUD, G., DE GOUYON, B., LAUTHIER, V., SILVER, L.M., DAUSSET, J., CANN, H.M. & SPIELMAN, R.S. (1992). Genetic mapping of three human homologues of murine t-complex genes localizes TCP10 to 6q27, 15cM distal to TCP1 and PLG. *Genomics*, **12**, 826-828.

BOYLE, J.M., HEY, Y., MYERS, K., STERN, P.L., GRZESCHIK, F.-H., IKEHARA, Y., MISUMI, Y. & FOX, M. (1992). Regional localisation of a trophoblast antigen-related sequence and 16 other sequences to human chromosome 6 using somatic cell hybrids. *Genomics*, **12**, 693-698.

CAVENEE, W.B., DRYJA, T.P., PHILLIPS, R.A., BENEDICT, W.F., GODBOUT, R., GALLIE, B.L., MURPHREE, A.L., STRONG, L.C. & WHITE, R.L. (1983). Expression of recessive alleles by chromosomal mechanisms in retinoblastoma. *Nature*, **305**, 779-784.

DEVILEE, P., VAN VLIET, M., VAN SLOUN, P., KUIPERS DIJKSHOORN, N., HERMANS, J., PEARSON, P.L. & CORNELISSE, C.J. (1991). Allelotype of human breast carcinoma: a second major site for loss of heterozygosity is on chromosome 6q. *Oncogene*, **6**, 1705-1711.

EHLEN, T. & DUBEAU, L. (1990). Loss of heterozygosity on chromosomal segments 3p, 6q and 11p in human ovarian cancer. *Oncogene*, **5**, 219-223.

FEINBERG, A.P. & VOGELSTEIN, B. (1983). A technique for radiolabeling DNA restriction endonuclease fragments to high specific activity. *Analyt. Biochem.*, **132**, 6-13.

FOX, H. (1990*a*). The pathology of early malignant change. In *Ovarian Cancer. Biological and Therapeutic Challenges*, Sharp, F., Mason, W.P. & Leake, R.E. (eds) pp. 165-167. Chapman and Hall: London.

FOX, H. (1990*b*). Malignant potential of benign ovarian cysts: the case 'against'. In *Ovarian Cancer. Biological and Therapeutic Challenges*, Sharp, F., Mason, W.P. & Leake, R.E. (eds), pp. 185-186. Chapman and Hall: London.

FRIEND, S.H., BERNARDS, R., ROGELJ, S., WEINBERG, R.A., RAPAPORT, J.M., ALBERT, D.M. & DRYJA, T.P. (1986). A human DNA segment with properties of a gene that predisposes to retinoblastoma and osteosarcoma. *Nature*, **323**, 643-646.

FUTREAL, P., SÖDERKVIST, P., MARKS, J.R., IGLEHART, J.D., COCHRAN, C., BARRETT, J.C. & WISEMAN, R.W. (1992). Detection of frequent allelic loss on proximal chromosome 17 in sporadic breast carcinoma using microsatellite length polymorphisms. *Cancer Res.*, **52**, 2624-2627.

GARCIA, T., SANCHEZ, M., COX, J.L., SHAW, P.A., ROSS, J.B.A., LEHRER, S. & SCHACTER, B. (1989). Identification of a variant form of the human estrogen receptor with an amino acid replacement. *Nucleic Acids Res.*, **17**, 8364.

GOELZ, S.E., HAMILTON, S.R. & VOGELSTEIN, B. (1985). Purification of DNA from formaldehyde fixed and paraffin embedded human tissue. *Biochem. Biophys. Res. Commun.*, **130**, 118-126.

KNUDSON, A.G. (1971). Mutation and cancer: A statistical study of retinoblastoma. *Proc. Natl. Acad. Sci.*, **68**, 820-823.

LE BORGNE-DEMARQUOY, F., KWIATOWSKI, T.J. & ZOGHBI, H.Y. (1991). Two dinucleotide repeat polymorphisms at the D6S202 locus. *Nucleic Acids Res.*, **19**, 6060.

LEE, J.H., KAVANAGH, J.J., WILDRICK, D.M., WHARTON, J.T. & BLICK, M. (1990). Frequent loss of heterozygosity on chromosomes 6q, 11 and 17 in human ovarian carcinomas. *Cancer Res.*, **50**, 2724-2728.

LITT, M. & LUTY, J.A. (1990). Dinucleotide repeat at the D6S89 locus. *Nucleic Acids Res.*, **18**, 4301.

MARKIE, D., FOULKES, W. & BODMER, W.F. (1992). Three probes recognise the same locus and form part of a linkage group on the long arm of chromosome six. *Cytogenet Cell Genet.*, **58**, 1914.

MAUVIEUX, V., JOUANOLLE, A.M., EL KAHLOUN, A., BLAYAU, M., LE GALL, J.Y. & DAVID, V. (1991). Dinucleotide repeat polymorphism at the FTHP1 locus on chromosome 6. *Nucleic Acids Res.*, **19**, 6969.

MCGUIRE, W.L., CHAMNESS, G.C. & FUQUA, S.A. (1992). The importance of normal and abnormal oestrogen receptor in breast cancer. In *Growth Regulation by Nuclear Hormone Receptors*. Cancer Surveys, Vol. 14. Parker, M.G. (ed.), pp. 31–40. Cold Spring Harbor Laboratory Press: New York.

MILLIKIN, D., MEESE, E., VOGELSTEIN, B., WITKOWSKI, C. & TRENT, J. (1991). Loss of heterozygosity for loci on the long arm of chromosome 6 in human malignant melanoma. *Cancer Res.*, **51**, 5449–5453.

MITELMAN, F. (1991). *Catalog of Chromosome Aberrations in Cancer*. 4th Edition, Wiley-Liss: New York.

MORITA, R., SAITO, S., ISHIKAWA, J., OGAWA, O., YOSHIDA, O., YAMAKAWA, K. & NAKAMURA, Y. (1991). Common regions of deletion on chromosomes 5q, 6q and 10q in renal cell cancer. *Cancer Res.*, **51**, 5817–5820.

MÜLLENBACH, R., LAGODA, P.J.L. & WELTER, C. (1989). An efficient salt-chloroform extraction of DNA from blood and tissues. *Trends Genet.*, **5**, 391.

OKA, K., ISHIKAWA, J., BRUNER, J.M., TAKAHASHI, R. & SAYA, H. (1991). Detection of loss of heterozygosity in the p53 gene in renal cell carcinoma and bladder cancer using the polymerase chain reaction. *Mol. Carcinog.*, **4**, 10–13.

PEJOVIC, T., HEIM, S., MANDHAL, N., BALDETORP, B., ELMFORS, B., FLODERUS, U.-M., FURGYIK, S., HELM, G., HIMMELMANN, A., WILLEN, H. & MITELMAN, F. (1992). Chromosomal aberrations in 35 primary ovarian carcinomas. *Genes Chromosom. Cancer*, **4**, 58–68.

PHILLIPS, R.A., DUNN, J., HAMEL, P., NOBLE, J., YOUNGSON, B., GILL, M., ZHENG, S., ZHU, X., COHEN, B.L., BECKER, A.J. & GALLIE, B.L. (1991). Retinoblastoma gene: mutations, expression, and putative function. In *Molecular Mechanisms and their Clinical Applications in Malignancies*. Bristol-Myers Squibb Cancer Symposia, Vol. 12. Bergsagel, D.E. & Mak, T.W. (eds), pp. 199–214. Academic Press: San Diego.

PONGLIKITMONGKOL, M., GREEN, S. & CHAMBON, P. (1988). Genomic organization of the human oestrogen receptor gene. *EMBO J.*, **7**, 3385–3388.

RANUM, L.P.W., CHUNG, M.-Y., DUVICK, L.A., ZOGHBI, H.Y. & ORR, H.T. (1991). Dinucleotide repeat polymorphism at the D6S109 locus. *Nucleic Acids Res.*, **19**, 1171.

RUSSELL, P. (1987). Common epithelial tumours of the ovary. In *Obstetrical and Gynaecological Pathology*, Third edition, Vol. 1, Fox, H. (ed.), pp. 556–622. Churchill Livingstone: Edinburgh.

RUSSELL, S.E.H., HICKEY, G.I., LOWRY, W.S., WHITE, P. & ATKINSON, R.J. (1990). Allele loss from chromosome 17 in ovarian cancer. *Oncogene*, **5**, 1581–1583.

SATO, T., TANIGAMI, A., YAMAKAWA, K., AKIYAMA, F., KASUMI, F., SAKAMOTO, G. & NAKAMURA, Y. (1990). Allelotype of breast cancer: cumulative allele losses promote tumor progression in primary breast cancer. *Cancer Res.*, **50**, 7184–7189.

SATO, T., SAITO, H., MORITA, R., KOI, S., LEE, J.H. & NAKAMURA, Y. (1991). Allelotype of human ovarian cancer. *Cancer Res.*, **51**, 5188–5122.

SEROV, S.F., SCULLY, R.E. & SOBRIN, L.H. (1973). Histological typing of ovarian tumours. In *International Histological Classification of Tumours*. Number 9. pp. 17–54. World Health Organization: Geneva.

SLEVIN, M.L. (1986). Ovarian Cancer. In *Randomised Trials in Cancer – A Critical Review by Sites*. Slevin, M.L. & Staquet, J. (eds), pp. 385–416. Raven Press: New York.

STENMAN, G., SANDROS, J., MARK, J. & EDSTRÖM, S. (1989). Partial 6q deletion in a human salivary gland adenocarcinoma. *Cancer Genet. Cytogenet.*, **35**, 153–156.

THOMAS, G.A. & RAFFEL, C. (1991). Loss of heterozygosity on 6q, 16q and 17p in human central nervous system primitive neuroectodermal tumors. *Cancer Res.*, **51**, 639–643.

TRENT, J.M., STANBRIDGE, E.J., MCBRIDE, H.Y., MEESE, E.U., CASEY, G., ARAUJO, D.E., WITOWSKI, C.M. & NAGLE, R.B. (1990). Tumorigenicity in human melanoma cell lines controlled by introduction of human chromosome 6. *Science*, **247**, 568–571.

VOGELSTEIN, B., FEARON, E.R., KERN, S.E., HAMILTON, S.R., PREISINGER, A.C., NAKAMURA, Y. & WHITE, R. (1989). Allelotype of colorectal carcinoma. *Science*, **244**, 207–211.

WAKE, N., HRESCHCHYSHYN, M.M., PIVER, S.M., MATSUI, S.-I. & SANDBERG, A.A. (1980). Specific cytogenetic changes in ovarian cancer involving chromosome 6 and 14. *Cancer Res.*, **40**, 4512–4518.

WEBER, J.L., KWITEK, A.E., MAY, P.E. & ZOGHBI, H.Y. (1991). Dinucleotide repeat polymorphism at the D6S105 locus. *Nucleic Acids Res.*, **19**, 968.

WEINBERG, R.A. (1991). Tumor suppressor genes. *Science*, **254**, 1138–1146.

ZHENG, J., ROBINSON, W.R., EHLEN, T., YU, M.C. & DUBEAU, L. (1991). Distinction of low grade from high grade human ovarian carcinomas on the basis of losses of heterozygosity on chromosomes 3, 6 and 11 and HER-2/*neu* gene amplification. *Cancer Res.*, **51**, 4045–4051.

ZOGHBI, H.Y., BALLANTYNE, C.M., O'BRIEN, W.E., MCCALL, A.E., KWIATKOWSKI, T.J., LEDBETTER, S.A. & BEAUDET, A.L. (1990). Deletion and linkage mapping of eight markers from the proximal short arm of chromosome 6. *Genomics*, **6**, 352–357.

ZUPPAN, P., HALL, J.M., LEE, M.K., PONGLIKITMONGKOL, M. & KING, M.-C. (1991). Possible linkage of the estrogen receptor to breast cancer in a family with late-onset disease. *Am. J. Hum. Genet.*, **48**, 1065–1068.